# An RNA Interference Phenotypic Screen Identifies a Role for FGF Signals in Colon Cancer Progression

**DOI:** 10.1371/journal.pone.0023381

**Published:** 2011-08-11

**Authors:** Marc Leushacke, Ralf Spörle, Christof Bernemann, Antje Brouwer-Lehmitz, Johannes Fritzmann, Mirko Theis, Frank Buchholz, Bernhard G. Herrmann, Markus Morkel

**Affiliations:** 1 Department of Developmental Genetics, Max Planck Institute for Molecular Genetics, Berlin, Germany; 2 Department of Biology, Chemistry and Pharmacy, Free University Berlin, Berlin, Germany; 3 Max Delbrück Center for Molecular Medicine, Berlin, Germany; 4 Department of Surgery, Heidelberg University Hospital, Heidelberg, Germany; 5 Max Planck Institute of Molecular Cell Biology and Genetics, Dresden, Germany; 6 University Hospital Carl Gustav Carus and Medical Faculty, University of Technology Dresden, Dresden, Germany; 7 Institute for Medical Genetics, Charité Campus Benjamin Franklin, Berlin, Germany; Technische Universität München, Germany

## Abstract

In tumor cells, stepwise oncogenic deregulation of signaling cascades induces alterations of cellular morphology and promotes the acquisition of malignant traits. Here, we identified a set of 21 genes, including *FGF9*, as determinants of tumor cell morphology by an RNA interference phenotypic screen in SW480 colon cancer cells. Using a panel of small molecular inhibitors, we subsequently established phenotypic effects, downstream signaling cascades, and associated gene expression signatures of FGF receptor signals. We found that inhibition of FGF signals induces epithelial cell adhesion and loss of motility in colon cancer cells. These effects are mediated via the mitogen-activated protein kinase (MAPK) and Rho GTPase cascades. In agreement with these findings, inhibition of the MEK1/2 or JNK cascades, but not of the PI3K-AKT signaling axis also induced epithelial cell morphology. Finally, we found that expression of *FGF9* was strong in a subset of advanced colon cancers, and overexpression negatively correlated with patients' survival. Our functional and expression analyses suggest that FGF receptor signals can contribute to colon cancer progression.

## Introduction

Late steps of tumor progression can result in metastatic dissemination of tumor cells, which have lost epithelial characteristics and acquired migratory and invasive capabilities. Morphologically, this phenotypic switch resembles the conversion of stationary epithelial cells into migratory mesenchymal cells during embryonic development, and therefore all of these processes are frequently termed epithelial-to-mesenchymal transition (EMT) [1], [2], [3], [4], [5]. Key steps in developmental and tumor-associated EMT are loss of E-Cadherin-based cell adhesion (by epigenetic silencing or transcriptional repression of *CDH1*, coding for E-Cadherin), reorganisation of the cytoskeleton leading to altered cell morphology, and induction of mesenchymal-specific gene expression, such as activation of *FN1* and *VIM* [6], [7], [8], [9], [10], [11]. In the embryo, EMT occurs in an ordered and highly reproducible manner. In tumor cells, however, EMT-like phenomena can be temporary, chaotic and incomplete.

In the embryo, EMT is induced by coordinated activation of signaling pathways, while in tumor cells complete or partial EMT can be elicited by the combined activities of oncogenic mutations and growth factor signals received from within the tumor cell microenvironment. Still, roles of key signaling pathways and molecules appear to be conserved. For instance, β-Catenin is required for the first embryonic EMT leading to formation of the primitive streak and mesoderm [12], while in colon carcinoma and other tumors β-Catenin transduces key signals at the invasive front, leading to loss of tumor cell adhesion, induction of cell motility and metastasis [13], [14], [15]. Multiple receptor tyrosine kinases (RTK) have been implicated in both, developmental and tumor-associated EMT. In embryonic development, the scatter factor/hepatocyte growth factor receptor MET is essential for migration of muscle progenitor cells, the epidermal growth factor (EGF) receptor is required for epithelial morphogenesis [16], [17], [18], and the fibroblast growth factor (FGF) receptor Fgfr1 and its ligand Fgf8 are essential for cell migration through the primitive streak and mesoderm formation [19], [20]. In tumors, unphysiological signaling through RTKs such as MET, the vascular endothelial growth factor (VEGF) receptor, and the EGF and FGF receptors can be associated with progression and metastasis [21], [22]. Importantly, the EGF and VEGF receptors are targets for rational therapy of advanced colon cancer [23].

Mitogen-activated protein kinase (MAPK) pathways, such as the Jun-N-terminal-kinase (JNK) and the mitogen-activated protein kinase kinase/extracellular-regulated kinase (MEK/ERK) pathways, integrate signals from multiple RTKs to control cell proliferation, cell motility and other cellular traits [24]. Oncogenic mutations in genes coding for signal transducers that relay signals from RTKs to MAPK pathways such as *KRAS* or *BRAF* can sensitize signaling via the MEK/ERK pathway in tumors, and such mutations invalidate therapeutic intervention strategies at upstream RTK receptors [25]. Both, during development and in tumors, EMT signals are frequently integrated by transcriptional repressors of *CDH1*, such as Snail, Snail2, Twist and others, which thus play important roles in the control of cell morphology and tumor progression [9], [10], [26].

Here, we took advantage of the molecular similarity between developmental and tumor-associated EMT to identify new candidate genes involved in colon cancer progression. In an RNA interference screen we identified multiple genes whose inactivation promoted an epithelial phenotype and E-Cadherin-mediated cell adhesion in SW480 colon cancer cells. Many of the genes identified code for components of key signaling pathways, such as the Wnt, Hedgehog and RTK pathways, for instance *FGF9*, *PTCH1*, *GLI2*, *GLI3* and *TCF7L1*. Using the identification of *FGF9* as a lead, we found that FGFR signals play roles in loss of colon cancer cell adhesion and induction of cell motility. To mediate these effects, FGFR signals modulate the MEK/ERK cascade and Rho GTPases. Our results thus identify FGFR signals as major determinants of the MEK/ERK cascade activity in SW480 colon cancer cells, even in the presence of an oncogenic KRAS mutation. We found that *FGF9* was expressed in colon cancer and expression levels correlate with patient survival, suggesting a functional role in the cancer.

## Results

### An RNAi interference phenotypic screen for genes that regulate colon tumor progression

**Figure 1:**
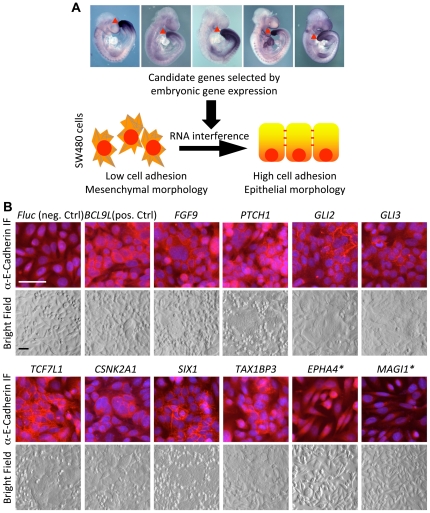
An RNA interference phenotypic screen in SW480 colon cancer cells. **A** Schematic overview of the screen: Genes were selected by screening the MAMEP database (http://mamep.molgen.mpg.de) for genes that display caudally restricted expression in the mouse embryo and literature searches. Example images of such gene expression patterns are displayed on top of the flow chart. Human homologues were inactivated by RNA interference in SW480 cells. Positive genes were identified by their ability to promote epithelial cell morphology. **B** E-Cadherin distribution and morphology of SW480 cells transfected with esiRNA against firefly luciferase (*Fluc*, negative control), *BCL9L* (positive control) and several positive genes. Genes marked with (*) induced a more spindle-form morphology. Scale bars represent 50 µm. For a full list of genes targeted in the screen, see Table S1. For a full list of genes that scored positive, see Table 1.

Invasive growth and metastasis of tumors involves loss of epithelial cell adhesion and gain of cell motility. We reasoned that genes whose products play roles in tumor progression may be enriched among those expressed in the caudal end of the mid-gestation mouse embryo, a place where epithelial cells undergo developmental EMT, i.e. a phenotypic switch to a mesenchymal state of low cell adhesion and high cell motility. We therefore used the MAMEP database (http://mamep.molgen.mpg.de) and literature searches to compile a list of genes expressed preferentially in the caudal end of the mid-gestation (E9.5) embryo, and identified their human homologues. Next, we targeted these genes using esiRNAs [27] in SW480 human colon cancer cells, which display a mesenchymal (“spindle-form”) morphology, are motile and have little cell adhesion. We screened for genes whose inactivation promoted epithelial (“cobblestone”) cell morphology and localization of the epithelial cell adhesion molecule E-Cadherin to cell-cell contacts (Fig. 1A). Out of 364 genes screened, silencing of 18 candidates induced epithelial morphology comparable to interference with the reference *BCL9L*, which is a transcriptional co-activator of β-Catenin [28]. We also found three genes whose inactivation promoted a more spindle-form cell morphology. In contrast, control-transfected cells did not display consistent morphological changes (Fig. 1B; for interference efficacy, see Fig. S1; for a complete list of targeted genes and positives, see Table S1 and Table 1, respectively).

**Table 1:** Genes identified as positives in the RNA interference phenotypic screen in SW480 cells.

Ensembl ID	Gene Symbol	Molecular Function
ENSG00000137936	BCAR3	Adaptor protein with RasGEF domain
ENSG00000101266	CSNK2A1	Serine/threonine kinase
ENSG00000157557	ETS2	Transcription factor
ENSG00000048828	FAM120A	unknown (RNA-binding)
ENSG00000102678	FGF9	Fibroblast growth factor receptor ligand
ENSG00000128683	GAD1	Glutamate decarboxylase
ENSG00000074047	GLI2	Transcription factor (Hedgehog signaling)
ENSG00000106571	GLI3	Transcription factor (Hedgehog signaling)
ENSG00000061273	HDAC7	Histone deacetylase
ENSG00000117114	LPHN2	G-Protein coupled receptor
ENSG00000139946	PELI2	Scaffolding protein (MAPK signaling)
ENSG00000117707	PROX1	Homeobox transcription factor
ENSG00000185920	PTCH1	Hedgehog receptor
ENSG00000104332	SFRP1	Secreted modulator of Wnt activity
ENSG00000126778	SIX1	Homeobox transcription factor
ENSG00000005513	SOX8	HMG-Box transcription factor
ENSG00000108392	TAX1BP3	unknown, may play roles in Rho and/or Wnt signaling
ENSG00000152284	TCF7L1	HMG-Box transcription factor (Wnt signaling)
ENSG00000122870	BICC1*	unknown (Putative RNA-binding)
ENSG00000116106	EPHA4*	Receptor tyrosine kinase
ENSG00000151276	MAGI1*	Scaffolding protein (Cell-cell adhesion)

Ensembl ID, Gene Symbol and molecular function of genes whose inactivation induced a more epithelial phenotype is given. Entries marked with (*) at the end of the list represent genes whose inactivation induced a stronger mesenchymal phenotype. Molecular function was retrieved using the Genecards database (http://www.genecards.org).

**Figure 2:**
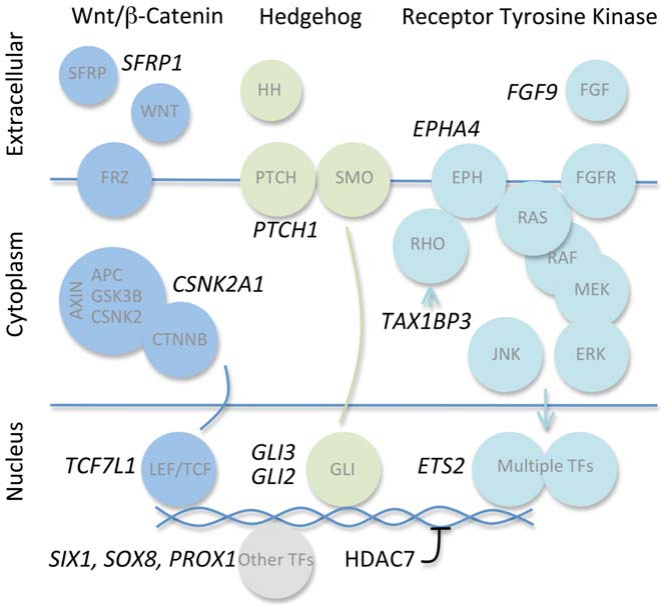
Pathway assignment of genes that scored positive in the RNA interference phenotypic screen. Gene symbols of positives are given in black letters, associated key pathway components are given in grey letters, displayed in colored circles and ordered by cellular compartment. Blue: Wnt pathway, Green: Hedgehog pathway, Cyan: RTK pathways. Genes were assigned to pathways using literature, Gene ontology and other database searches. Key references are given in the main text.

Many of the identified genes play known roles in the control of signaling pathways that regulate embryonic development, organ homeostasis, and tumor progression. We found *TCF7L1*, coding for a downstream transcription factor of the Wnt/β-Catenin signaling cascade [29], as well as the Wnt/β-Catenin signaling components *SFRP1* and *CSNK2A1*. We also isolated *PTCH1*, *GLI2*, and *GLI3*, which code for components of the Hedgehog signaling pathway [30]. We found *FGF9* and *EPHA4*, coding for an RTK ligand and a RTK of the ephrin subfamily regulating cell adhesion and the cytoskeleton via MAPK and Rho [31], [32]. The candidate gene *TAX1BP3* may also have roles in the regulation of Rho signals [33]. Among several genes implicated in the regulation of transcription, we found *SIX1*, coding for a homeobox transcription factor implicated in cell differentiation and tumor progression [34], [35] (Fig. 2).

### FGF signals regulate adhesion and motility of colon cancer cells

Since we identified *FGF9* in our cellular screen and found expression of FGF9 in intestinal malignancies of both mouse and humans (see below), we focused on the roles of FGF signals in colon carcinoma cells. First, we confirmed the screening result using an independent *FGF9* siRNA preparation, which likewise induced an epithelial phenotype in SW480, i. e. led to a loss of spindle-form cells and to an increase in membraneous E-Cadherin staining (Fig. S2). In another colon cancer cell line, HCT116, silencing of *FGF9* had no apparent effect. However, when we targeted *FGFR3*, coding for a high affinity receptor of FGF9 and other FGFs, we found that it promoted epithelial cell morphology in HCT116 (Fig. S2). These findings indicate that FGF receptor signals may commonly have roles in the control of colon cancer cell morphology, and that different pathway components may be limiting in the various cell lines.

**Figure 3:**
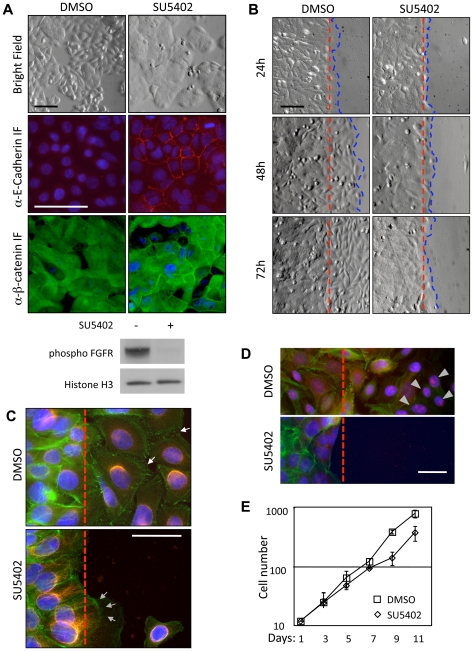
FGF receptor signals are essential for SW480 cell motility. **A** Phenotypic switch of SW480 cells upon FGF receptor inhibition by SU5402. Top to bottom: Cell morphology in bright field, immunostaining of E-Cadherin and β-Catenin. Photos were taken 72 h after start of SU5402 treatment. Below: SW480 cells receive FGF signals, which can be blocked by SU5402. **B** Scratch-wound motility assay upon FGF receptor inhibition. SW480 cells were grown to confluence, and scratches were induced 24 h after start of treatment. Bright field images of the scratch edge were taken 24, 48 and 72 hour after scratching. Scale bars in A and B represent 50 µm. **C**, **D** Immunostaining of the scratch edge. **C** Staining of the actin and the microtubule cytoskeleton in control and SU5402-treated cells. Green: actin (phalloidin); yellow: tubulin; blue: nuclei (DAPI); arrows: areas of dense focal adhesion in motile control cells; arrowheads: enhanced cortical actin staining in SU5402-treated cells. **D** RhoA redistribution in migrating cells, which is reduced in inhibitor-treated cells. Green: actin; red: RhoA; blue: nuclei. grey arrowheads mark nuclear RhoA. Dashed line marks scratch edge. Scale bars in C and D represent 25 µm. **E** Quantification of proliferation in control and SU5402-treated cells. For time-course of proliferation, cells were plated in replicates, and duplicate samples were counted bi-daily in a Neubauer hematocytometer.

Next, we targeted the FGF receptors using the small molecule inhibitor SU5402 [36], and assessed changes in the cellular phenotype. In agreement with our RNA interference data, inhibition of FGF receptors promoted epithelial morphology of SW480 cells and commanded E-Cadherin and β-Catenin to cell-cell contacts, indicating *de-novo* assembly of epithelial adherens junctions (Fig. 3A). Similar effects were observed on the morphology of SW620 cells, which are derived from the same patient, and on HT29 and HCT116 cells, which are derived from other patients (Fig. S3).

Cell adhesion and cell motility are often inversely correlated in tumor cells. We therefore also assessed cell motility. Control SW480 cells rapidly migrated, whereas SU5402-treated cells could no longer close a scratch wound (Fig. 3B). FGF receptor-inhibited cells at the scratch edge displayed increased staining of cortical actin and reduced numbers of focal adhesion contacts, indicating altered dynamics of the cytoskeleton (Fig. 3C). Furthermore, Rho GTPases, which are key effectors of the balance between cell adhesion and motility [37], appeared localized to nuclear speckles in SW480 cells invading the scratch, and were reduced in inhibitor-treated SW480 cells (Fig. 3D). Cell proliferation was only mildly affected by FGF receptor inhibition (Fig. 3E). We saw similar effects on cell morphology and motility using a structurally unrelated small molecule inhibitor of FGF receptors, PD173074 (data not shown). These results indicate that FGF receptor signals can play essential roles in the switch from a stationary to a migratory phenotype, which is a hallmark of the late steps of intestinal tumor progression resulting in metastasis.

### FGF signals in colon cancer cells are transmitted via the MAPK pathway and Rho

**Figure 4:**
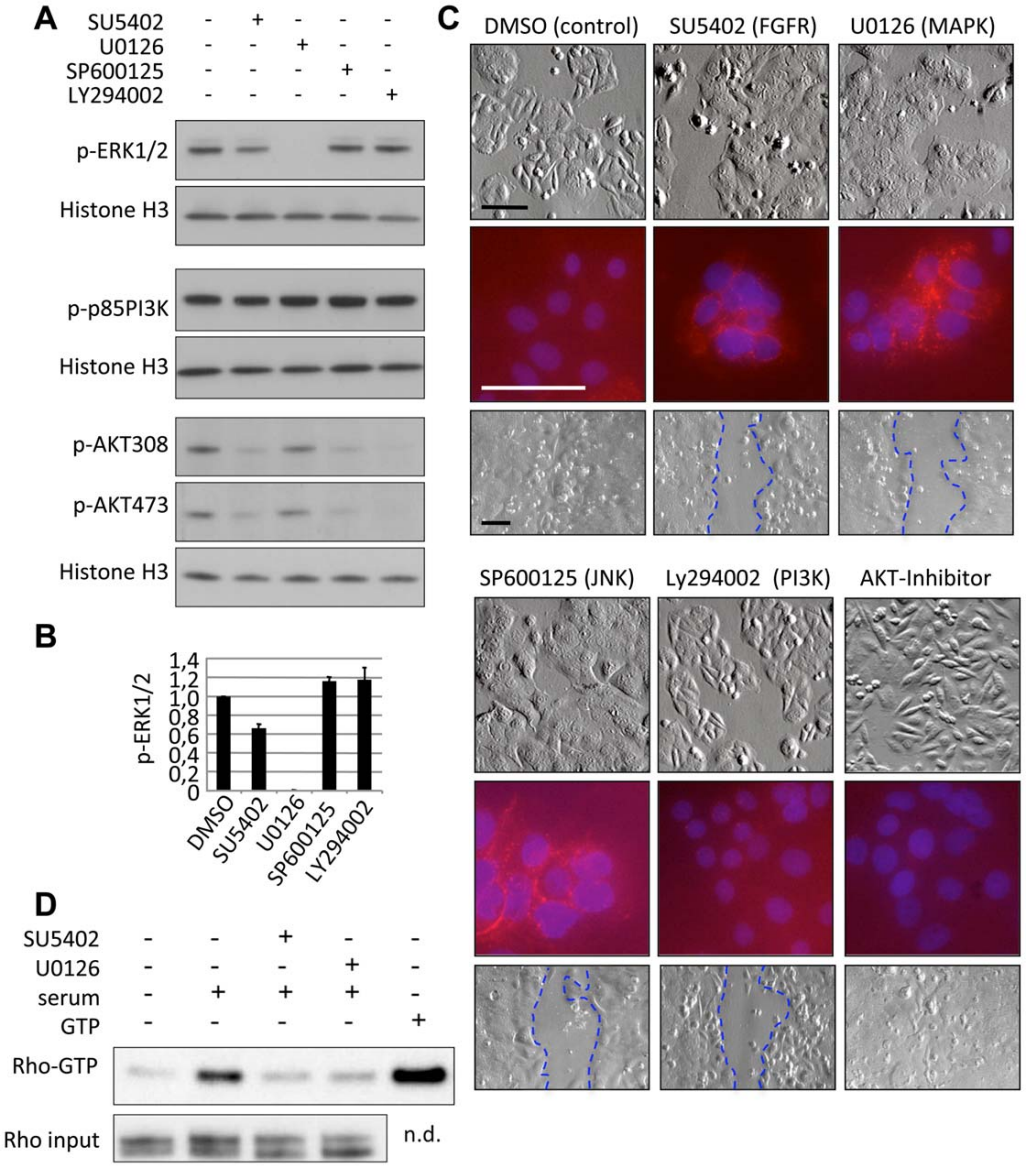
FGF receptor signals for epithelialization are transmitted via MEK1/2 and Rho. **A** Analysis of Phospho-ERK1/2, Phospho-P85PI3K and Phospho-AKT in SW480 cells after treatment with DMSO (solvent control), SU5402 (inhibiting FGFR), U0126 (inhibiting MEK1/2 dependent ERK1/2 phosphorylation), SP600125 (inhibiting JNK), and Ly294002 (inhibiting PI3K-mediated AKT phosphorylation) are shown. Histone H3 is shown as loading control. Phosphorylation status was assessed 40 minutes after inhibitor treatment. **B** Quantification of ERK1/2 phosphorylation, mean of three experiments. **C** Morphology, E-cadherin immunofluorescence and cell motility of SW480 cells after 72 h of inhibitor treatment. Inhibition of the classical MAPK or alternative MAPK/JNK cascades induced a similar phenotype as FGFR inhibition in SW480 cells, wheras inhibition of PI3K or AKT had no such effect. We found that PI3K inhibition, but not AKT inhibition, inhibits cell motility. Uncoupling of PI3K and AKT signals has been observed before in tumors [56]. Scratch assay was done as in Fig. 3B. Scale bars represent 50 µm. **D** Inhibition of FGF receptors or MEK1/2 leads to reduced Rho activity. Rho GTPase loading assays were performed in SW480 cells 5 min after serum stimulation in the presence or absence of SU5402 or UO126. N.d.: not determined.

FGF receptor signals are known to be transmitted via MAPK cascades and via the phosphoinositid-3-kinase (PI3K)/AKT signaling cascade [38]. We therefore analyzed the activity of key cellular signal transducers after inhibition of FGF receptors or key downstream signal transducers, i.e. U0126, inhibiting the MEK1/2 kinases of the classical MEK/ERK MAPK cascade, SP600125, an inhibitor of the alternative JNK cascade and LY294002, an inhibitor of PI3K signal transduction. As expected, inhibition of MEK1/2 abolished phosphorylation of ERK1/2, and inhibition of PI3K abolished phosphorylation of AKT. Upon FGF receptor inhibition, we found a limited, but reproducible reduction of ERK1/2 phosphorylation (of about 30%). In contrast, the activity of p85-PI3K was not affected. Changes in the phosphorylation of AKT indicated that additional signal transduction networks are modulated directly or indirectly when inhibiting FGF receptors or JNK (Fig. 4A, B).

We next tested phenotypic effects of inhibition of the downstream cascades. Treatment of SW480 cells with U0126 (inhibiting MEK1/2) or with SP600125 (inhibiting JNK) supported the formation of epithelial cell adhesion complexes, and blocked cell motility, similar to effects seen after inhibition of FGF receptor signals (Fig. 4C, Fig. S4). In contrast, phenotypes observed after treatment with LY294002 (inhibiting PI3K) or the Calbiochem AKT-Inhibitor were not comparable with effects seen after FGF receptor inhibition (Fig. 4C). A similar correlation of cellular phenotypes upon inhibition of signaling hubs was observed in HCT116 cells, which however did not display modulation of E-cadherin localization under any conditions (Fig. S5, see also Fig. S2). In summary, these results indicate that FGFR signals modulate activity of the MEK/ERK signaling cascade in SW480 cells, and that activity of FGFR, MEK1/2 and JNK are required for the maintenance of a phenotype that favors cell motility over cell adhesion.

Since we observed re-localization of Rho in motile SW480 cells (see above, Fig. 3D), we determined the impact of FGFR or MEK1/2 signals on Rho activity, using Rho loading assays. For this, we serum-starved SW480 cells for 24 h, and assessed Rho activation after serum re-stimulation. Addition of serum led to rapid induction of active Rho-GTP in control cells, while Rho activation was compromised in the presence of FGF receptor or MEK1/2 inhibitors (Fig. 4D). This result suggests that in SW480 cells FGF receptors and MEK1/2 play roles in the regulation of Rho GTPases, which are central in modulation of the cytoskeleton.

### FGFR and MAPK signals are associated with specific gene expression signatures

To isolate gene expression signatures related to FGFR signals and cell motility in colon cancer cells, we compared gene expression profiles of solvent control and inhibitor-treated SW480 cells. We first used functional clustering of genes deregulated between control (DMSO-treated) and SU5402-treated cells using Ingenuity pathway analysis (Ingenuity® Systems, www.ingenuity.com). Among the signaling networks most strongly affected, we identified a cluster of deregulated genes related to cell motility that centers on the ERK1/2-dependent MAPK pathway, corroborating our previous results (Fig. S6). The inferred network contains central signal transducers of motility signals such as *ERK1*/2 and *PTK2* (focal adhesion kinase), and converges on the transcription factors *JUN*/*JUNB* and *FOSL1*. Importantly, *FOSL1* has previously been identified as a key transcription factor in the control of tumor cell motility [39].

**Figure 5:**
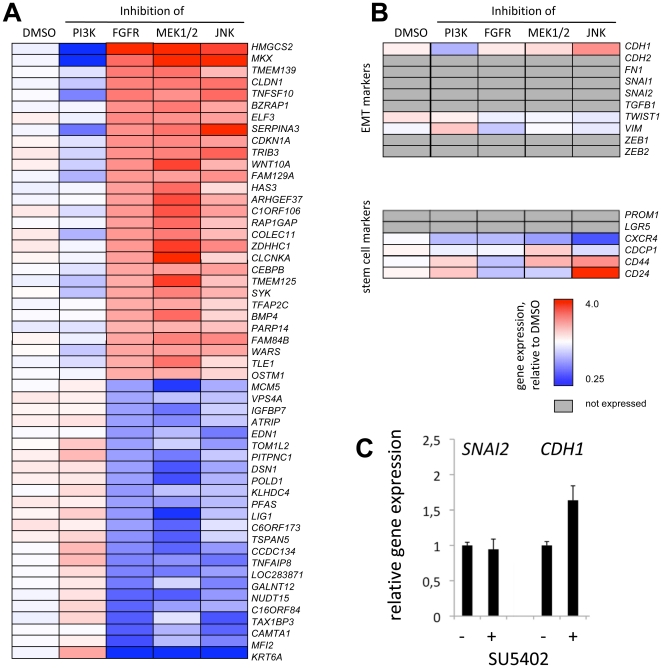
Epithelial and mesenchymal phenotypes of SW480 cells correlate with gene expression signatures. **A–B** Microarray analysis of SW480 after small molecular inhibition of FGFR, MEK1/2, JNK or PI3K. Average expression of triplicate experiments is displayed. Red: High expression; Blue: low expression White: Median expression across all profiles Grey: Genes with no or marginal expression **A** Clusters of genes whose activity correlates with epithelial and mesenchymal phenotypes. The following procedure was used to identify these genes: First, genes deregulated more than 1.3fold (p<0.01) after SU5402 treatment compared to control (DMSO) were isolated. Second, these genes were clustered in self-organizing maps, and three clusters that correlated with the phenotype were selected. Genes were ordered by fold-change expression after FGFR inhibition. **B** Assessment of key genes implicated in EMT or stemness. **C** Quantitative Real-time PCR analysis of *SNAI2* and *CDH1* after SU5402 (FGFR Inhibitor) treatment. Expression was normalized to solvent control samples.

Next, we compared DMSO-treated and PI3K-inhibitor-treated SW480 cells (displaying mesenchymal “spindle-form” morphology and weak cell adhesion) with FGFR-inhibitor-, MEK1/2-inhibitor and JNK-inhibitor treated cells (displaying epithelial “cobblestone” morphology and strong cell adhesion, see also Fig. 4C). We used self-organizing maps to identify groups of genes whose expression is associated with these distinct morphologies, and identified 29 genes which were associated with strong cell adhesion and 23 genes associated with weak cell adhesion. Signature genes of SW480 cells that have strong cell adhesion were *RAP1GAP*, which is involved in the assembly of adherens junctions and *CLDN1*, a component of adherens junctions, providing evidence that epithelial cell adhesion is regulated at least partly on the transcriptional level. We also found enhanced expression of the transcription factor genes *ELF3* and *HMGCS2*, which play functional roles in epithelial differentiation [40], [41], the tumor suppressors *SYK* and *TFAP2C* and the Rho regulator *ARHGEF37*. Signature genes of SW480 cells that have weak cell adhesion and mesenchymal morphology were enriched for genes that have been assigned oncogenic activity, namely *EDN1*, *IGFBP7*, *MCM5*, *MFI2* and *TNFAIP8*. Finally, we also found *TAX1BP3*, which we above isolated in our phenotypic esiRNA screen, suggesting a functional role for this gene in the regulation of adhesion and motility (Fig. 5A) [33]. We observed a similar pattern of gene regulation upon FGF receptor inhibition in HCT116 colon cancer cells (which displayed loss of spindle form upon *FGFR3* RNA interference or FGF receptor inhibition, see Fig. S2, S3), implicating a conserved role of this gene cluster downstream of the FGF receptors (Fig. S7).

When we assessed the expression of key modulators of EMT, we saw a small, albeit not significant, variation in the expression of *CDH1*, indicating that the more drastic changes that we see in E-Cadherin localization (see Fig. 1B, 3A) occur predominantly via posttranscriptional regulation. Expression of *VIM* and *TWIST* were only weakly affected, while other EMT markers were found to be non-expressed. These data suggest that SW480 cells do not undergo a complete mesenchymal-to-epithelial transition after FGF receptor or MAPK inhibition. We also did not see regulation of intestinal stem cell markers, which have recently been linked to EMT in tumors (Fig. 5B) [42], [43], [44]. Regulation of the transcriptional repressor *SNAI2* and its target gene *CDH1* via ERK1/2 signals has been demonstrated before in SW480 cells [45]. We therefore assessed the expression of *SNAI2* and *CDH1* by qRT-PCR, which can be more sensitive than array analysis. We could confirm the mild activation of *CDH1* after SU5402 treatment, while *SNAI2* was unchanged (Fig. 5C).

### FGF9 expression is correlated with patient's survival in human colon cancer

**Figure 6:**
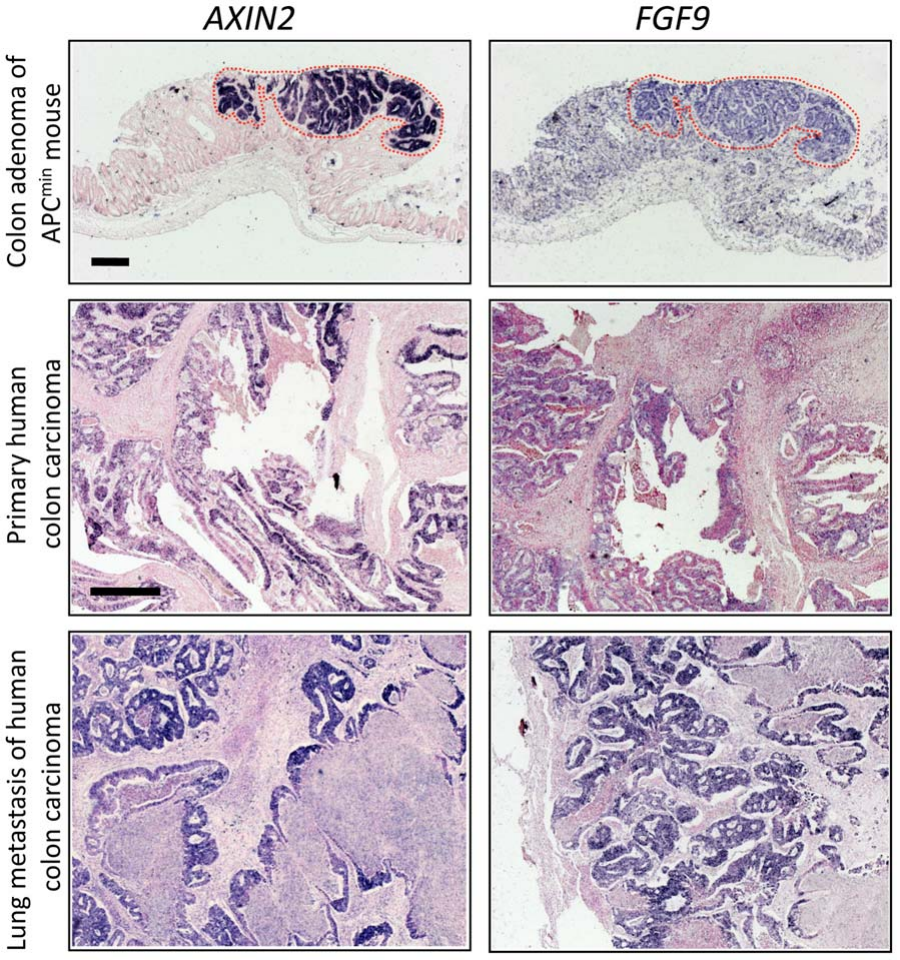
*FGF9* is expressed in mouse and human intestinal malignancies. Expression of the Wnt target gene *Axin2/AXIN2* (to the left) and *Fgf9*/*FGF9* (to the right) in mouse and human intestinal malignancies was determined by RNA *in-situ* hybridization. Upper panels: Adenoma of the colon from an APC^min^ mouse. Middle panels: primary human colon carcinoma. Lower panel: lung metastasis of human colon carcinoma. Negative control hybridization (using an unrelated asRNA probe of similar size and CG content, not shown) did not produce a signal. Scale bars represent 500 µm.

**Figure 7:**
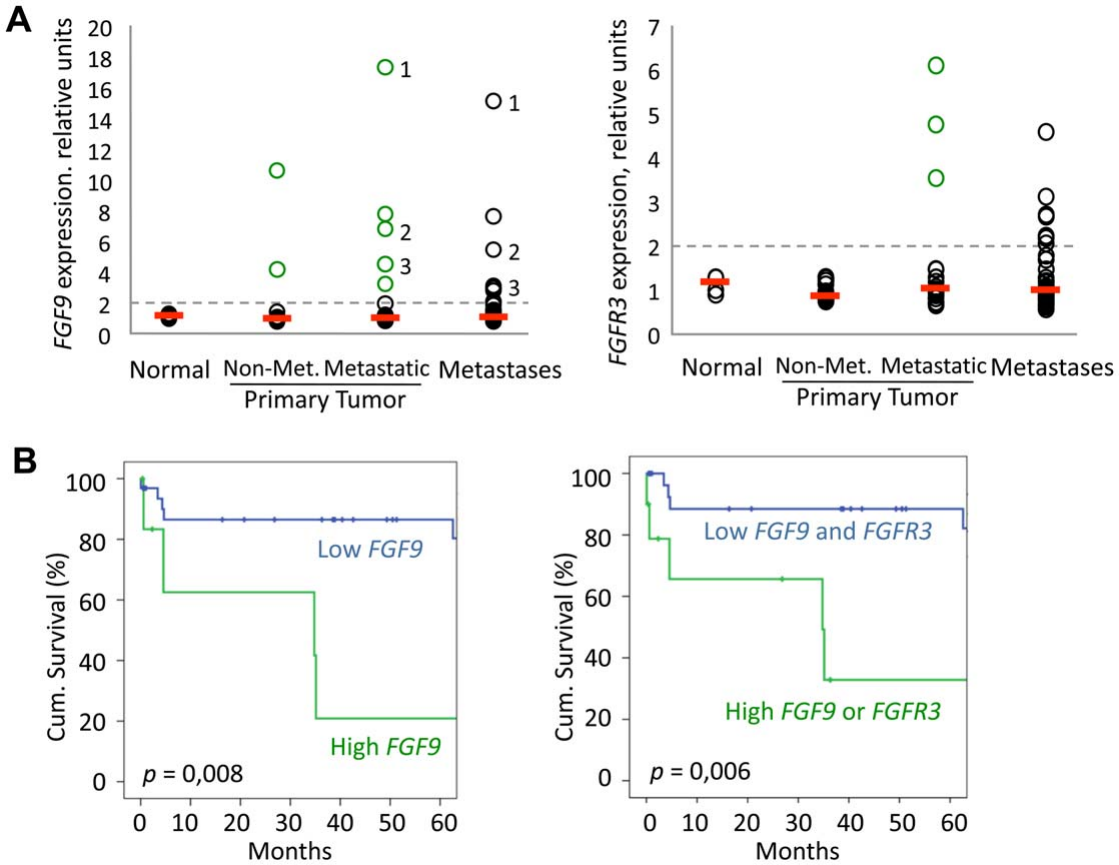
*FGF9* expression in colon carcinoma correlates with patients' survival. **A** Expression of *FGF9* or *FGFR3* in invasive and metastatic colon carcinoma, as assessed by microarray analysis (n = 95). Expression in each sample is indicated by circle, median expression in each group is given by red bar. Numbers in *FGF9* graph indicate primary tumor/metastasis pairs derived from the same patient. **B** Kaplan-Meier survival analysis of patients examined in A (primary tumors with follow-up data only, n = 39). Patients were grouped according to high (>2-fold of median, indicated by green circle in Fig. 7A) and low *FGF9* and *FGFR3* expression.

To assess the *in-vivo* significance of our analyses, we determined expression of *FGF9* in specimen of mouse and human intestinal tumors. In mouse intestinal adenoma, we found increased expression of *Fgf9* in a similar pattern as the Wnt target gene *Axin2*. When we determined expression of *FGF9* in human colon cancer, we likewise found generalized expression in the tumor epithelium in primary tumors and lung metastases (Fig. 6). Since our screen, in conjunction with our subsequent functional analysis of overall FGF signals, indicated a possible role for FGF9 signals in intestinal carcinoma, we examined the relative expression levels of *FGF9* in a set of 95 expression profiles of human colon cancer, comprising normal colon (4 profiles), advanced colon carcinoma (staged T3/4 by International Union Against Cancer (UICC) criteria; 41 profiles), and metastases to lymph nodes, liver and lung (50 profiles) [46]. We found basal expression levels of *FGF9* in the normal colon tissue profiles and in a majority of tumor profiles, but high expression of *FGF9* in a subgroup of 7 colon tumors and 5 metastases (Fig. 7A; cut-off>2-fold compared to normal tissue). Of note, we also found increased expression of *FGFR3*, coding for a high-affinity FGF9 receptor, in 3 primary tumor profiles and several metastases (Fig. 7A). The high expression of *FGF9* was maintained between pairs of primary tumors and metastases derived from three patients, indicating that *FGF9* activation in the tumor can persist over long periods of time.

We finally correlated the expression of *FGF9* in primary tumors (n = 39) with patient survival, using Kaplan-Meier analysis. The subgroup of 7 patients with high expression of *FGF9* had significantly shorter survival compared to patients with low *FGF9* expression (Fig. 7B, *p* = 0.008). We saw a similar result, when we included the patients with high expression of *FGFR3* in the group (Fig. 7B, *p* = 0.006). The observed correlation between high expression of *FGF9* and short patient survival may indicate functional roles of FGF9 in tumor progression.

## Discussion

Here, we undertook a focused screen to identify genes whose expression is required to maintain a mesenchymal, potentially metastatic phenotype in SW480 colon cancer cells, and isolated multiple candidates. Many of these genes can be linked to major signaling pathways or have been implicated in EMT and tumor progression before. We found the HMG-box transcription factor gene *TCF7L1* (also known as *TCF3*), which can transmit Wnt/β-Catenin signals [29]. Interestingly, the related transcription factor TCF7L2 (also known as TCF4) is required to transmit the Wnt/β-Catenin signal and to maintain the stem cell compartment in the intestine [47], [48]. The family member *TCF7L1* is however also expressed in the colon crypt and in colon cancer (Fig. S8). Our results therefore indicate that *TCF7L1* may have an as yet unidentified role in transmission of tumor-related β-Catenin signals.

We also identified *PTCH*, *GLI2* and *GLI3*, which code for components of the Hedgehog signaling pathway. Possible roles of Hedgehog signals in colon cancer progression and metastasis are controversial [49], [50]. Results of our screen support a role of Hedgehog signals in the modulation of cell adhesion in colon cancer cells.

Unexpectedly, the screen yielded also three genes whose inactivation promoted a more spindle-form phenotype. Two of these, *BICC1* and *MAGI1* have been shown before to be required for the assembly of E-Cadherin-mediated cell adhesion complexes [51], [52]. The finding of functional roles of these genes in the maintenance of residual cell-cell contacts in tumor cells may indicate a more universal role for these genes in the regulation of cell adhesion than anticipated before.

FGF signals have been implicated in the control of intestinal cell motility and colon cancer progression before. For instance, FGF1 and FGF2 have been shown to promote migration of intestinal epithelial cells [53], and specific isoforms of FGFR3 can mediate growth and migration of colon cancer cells [54]. In our screen, we identified *FGF9* as a gene implicated in the regulation of colon cancer cell adhesion. Using small molecular inhibitors and activity assays of key signal transducers, we found that the MAPK cascade and Rho GTPases are critical in the transmission of FGF signals that control cell adhesion and motility in colon cancer cells, while the PI3K cascade is not. SW480 colon cancer cells contain a well-characterized set of mutations, including an activating *KRAS* (G12V) mutation [55]. Our data therefore imply that oncogenic mutations in KRAS do not fully uncouple MAPK activity from upstream stimuli, but may instead potentiate RTK signal transduction. In the patient, therapy success using VEGF and EGF receptor inhibitors depends crucially on the absence of downstream KRAS or BRAF mutations [25].

Recent models of tumor progression imply an uncontrolled activation of developmental signaling pathways and embryonic gene expression programs, resulting in a coordinated loss of cell adhesion, gain of cell motility and the acquisition of stem cell traits in metastatic tumor cells [42], [43], [44]. Our screen identifies multiple candidate genes that could play roles in these processes. We furthermore provide evidence that FGF receptor signals in colon tumor cells can promote pro-metastatic traits by skewing the balance between cell adhesion and cell motility. In line, we found that expression of *FGF9* in human colon cancer is correlated with patient's survival in the limited set of tumors that we examined. Further studies are required to assess whether *FGF9* could serve as a prognostic factor in colon carcinoma and whether gene expression programs activated by FGF9 are implicated in the regulation of tumor cell adhesion, motility and metastasis in intestinal cancer.

## Materials and Methods

### esiRNA screen

Human homologues of the selected MAMEP mouse gene entries were identified using the HomoloGene (NCBI) and Ensembl (EMBL) databases, and esiRNA preparations were produced for these genes, as described before [27]. SW480 cells were obtained from American Type Culture Collection and propagated in DMEM (Invitrogen), 10% fetal bovine serum. For screening of candidate genes, SW480 cells were plated at a density of 3×10^4^ cells/cm^2^ onto glass cover slips, and esiRNAs were transfected in 48-well plates using Oligofectamine (Invitrogen), essentially as described previously [27]. esiRNAs against Firefly luciferase and *BCL9L* were used as negative and positive controls, respectively, in each plate of the screen. Representative bright field and E-cadherin immunofluorescence (see below) images were taken 72 h after transfection, and judged independently of each other. Images that displayed unusually high cell densities were removed from the analysis. Genes were assigned positives when they scored in both, the bright field and the immunofluorescence image screen.

### Cell-based assays

All colon cancer cell lines were propagated in DMEM, 10% fetal bovine serum. For scratch wound assays, cells were plated at a density of 3×10^5^/cm^2^. The following inhibitors were employed: SU5402 (42 µM), PD173047 (60 µM), U0126 (10 µM), SP600125 (20 µM), Ly294002 (20 µM), and AKT-Inhibitor (30 µM, Calbiochem). For immunostaining, cells were washed with PBS, fixed in 4% formaldehyde and permeabilized with 0.5% Triton X-100. The following antibodies and reagents were used: α-E-Cadherin, α-β-Catenin (BD), α-RHOA, α-tubulin (Santa Cruz), phalloidin-FITC (Invitrogen). For Western blotting, the following antibodies were employed: α-pERK1/2, α-pAKT, α-pPI3K, α-pFGFR (Cell Signal). For assessment of FGF signaling in SW480, cells were transfected with a plasmid coding for FGFR1, since the pFGFR1 antibody employed cannot detect endogenous receptor activity, according to the manufacturer. For Rho activity assays, SW480 were seeded at 5×10^4^ cells/cm^2^, and serum-starved for 24 h. Rho activity was assessed 5 min after serum stimulation, using a Rho activation assay kit (Millipore/Upstate; α-RhoA,B,C antibody) according to manufacturer's protocol. Western blot signals were detected using chemoluminescence (GE Healthcare) and quantified using ImageJ software (http://rsbweb.nih.gov/ij). qRT-PCR was done using random-hexamer primed cDNA prepared according to manufacturers' protocols (Invitrogen), the Step One Plus detection system and SYBR green. Data was analyzed with Step One Software v2.1 (Applied Biosystems) using the CT(ΔΔCT) method. Marker expression was normalized to *GAPDH* as a reference gene. Primer sequences are available upon request.

### Microarray analysis

For microarray analysis, SW480 were treated with DMSO (solvent control), FGF receptor inhibitor (SU5402), MAPK Inhibitor (UO126), JNK inhibitor (SP600125), or PI3K inhibitor (Ly294002) for 24 h as above, using three replicates per treatment group. RNA was isolated using RNeasy columns (Qiagen), labeled using the MessageAMP Kit (Ambion), and hybridized to Illumina Sentrix Hu8-v3 bead chips according to manufacturer's instructions. Expression data was processed using Genome Studio GX software (Illumina), using the cubic spline normalization method and background subtraction. Non-expressed and marginally expressed genes were removed for analysis by a detection value cut-off of 1 in at least 6 out of 15 profiles. Genes were grouped by self-organising map cluster analysis and visualized using MultiExperiment Viewer (http://www.tm4.org). Microarray data are available via GEO accession number GSE29689.

### Histology

For *in-situ* hybridization, tissues were fixed in 4% formaldehyde, dehydrated via a graded ethanol series, embedded in paraffin, and sectioned at 4 µm. *In-situ* hybridization was performed as described before [46]. Primer sequences flanking the *in-situ* probes are available upon request.

## Supporting Information

Figure S1 **esiRNA treatment induces effective mRNA interference in SW480 cells.** SW480 cells were transfected with esiRNAs directed against selected genes or with control esiRNA, and mRNA levels were assessed 48 h after transfection, using qRT-PCR. Expression of experimental genes was normalized versus GAPDH. Residual gene expression after specific esiRNA treatment is given relative to control transfections. Bars indicate mean of three technical replicates and standard error.
(TIF)

Figure S2 **Phenotypic analysis of SW480 and HCT116 colon cancer cells after FGF9 or FGFR3 mRNA interference.** A RNA interference with FGF9 cells leads to loss of spindle form and increased membraneous E-cadherin staining in SW480, confirming the screening result (see Fig. 1B). Silencing of FGFR3 does not induce a visible phenotype in SW480. B Interference with FGFR3, but not with FGF9, results in loss of spindle form in HCT116 (see Bright Field), however not to an increase in membraneous E-cadherin. SW480 and HCT116 were transfected with Dharmacon smart pool siRNAs, according to manufacturer's instructions, using DharmaFECT1 and a final concentration of 25 nM siRNA. Scale bars 50 µm.
(TIF)

Figure S3 **Multiple colon cancer cell lines re-epithelialize upon FGF receptor inhibition.** Phenotypes of a panel of colon cancer cell lines after FGF receptor inhibition, using SU5402. SW620, HT29 and HCT116 cells show aspects of re-epithelialization, such as loss of spindle form or disappearance of visible cell-cell contacts. In contrast, DLD1 (lower panels), WiDr, Caco2 and Cx1 cells (not shown) do not show visible phenotypic alterations after inhibitor treatment. Images were taken 72 h after start of the treatment, as in Material and Methods. Scale bar represents 50 µm.
(TIF)

Figure S4 **Inhibition of MEK1/2 by U0126 induces re-epithelialization and blocks cell motility in SW480 cells.** Left: Phenotypic switch of SW480 cells upon MEK1/2 inhibition. Top to bottom: Cell morphology in phase contrast, immunofluorescence of E-cadherin and b-Catenin, which are components of adherens junctions. Photos were taken 72 h after start of the treatment. Right: Scratch-wound motility assay upon MEK1/2 inhibition. SW480 cells were grown to confluence, and scratches were induced 24 h after start of inhibitor treatment. Photos show phase-contrast images of the scratch edge and were taken 24, 48 and 72 hour after scratching. Red line indicates scratch edge, blue line indicates cell migration front. Scale bars represent 50 µm.
(TIF)

Figure S5 **Phenotypic effects of inhibitor treatment in HCT116 colon cancer cells.** Scratch-wound assays were performed upon inhibition of FGF receptors, MEK1/2, JNK or AKT, as indicated. HCT116 cells were grown to confluence, and scratches were induced 24 h after start of treatment. Solvent control-treated cells, as well as AKT- inhibitor-treated cells close the scratch wound, whereas FGFR, MEK1/2 or JNK inhibitor treatments lead to a delay in scratch wound closure. Inhibition of PI3K leads to marked loss of cells at the scratch edge (not shown). Bright field images of the scratch were taken 72 hour after scratching. Red line indicates scratch edge, blue line indicates cell migration front. Scale bar represents 100 µm.
(TIF)

Figure S6 **Ingenuity pathway analysis identifies a network implicated in cell motility, which is enriched in FGF-regulated genes.** Green: downregulated genes after SU5402 treatment, red: upregulated genes after SU5402 treatment. Grey: non-regulated genes within the network. Network was derived from genes deregulated (>1.3-fold, p<0.05) in expression profiles from biological triplicates of SU5402-treated SW480 cells and solvent controls. Profiles were normalized in Illumina Genome Studio using cubic spline normalization and background subtraction. Absent and marginally expressed genes were removed before analysis.
(TIF)

Figure S7 **An SW480-derived gene expression program associated with epithelial and mesenchymal morphology is also modulated in HCT116 cells.** Analysis of gene expression in HCT116, 24 h after treatment with SU5402 or in solvent control (DMSO). Figure shows genes whose expression has previously been identified to correlate with mesenchymal or epithelial phenotypes in SW480 cells (see Fig. 5A). Genes that are not expressed in HCT116 were removed. Gene expression profiles were assessed using Illumina Sentrix Hu8-v2 bead chips according to manufacturer's instructions. Data was processed from biological duplicates using Illumina Genome Studio, as detailed in the Material and Methods section. Red indicates high expression; blue low expression and white average expression.
(TIF)

Figure S8 ***TCF7L1*** **expression is delocalized and upregulated in human colon carcinoma.** Expression of *TCF7L1* in normal human colon (upper panel), primary human colon carcinoma (middle panel), and lung metastasis of human colon carcinoma (lower panel), as assessed by RNA in-situ hybridization. Negative control hybridization (using an unrelated asRNA probe of similar size and CG content) did not produce a signal (not shown).
(TIF)

Table S1 **Full list of genes that were targeted by esiRNA in the loss-of-function phenotypic screen.** Table gives Gene Symbols, ENSEMBL IDs, esiRNA information and comments on screening results. Bold Gene Symbols correspond to current ENSEMBL IDs (GRCh37.p3, release 62), Gene Symbols in regular type may correspond to previous builds.
(XLS)
