# Supplementary figures and images for: An RNA Interference Phenotypic Screen Identifies a Role for FGF Signals in Colon Cancer Progression
Source: PLoS One. 2011 Aug 11;6(8):e23381. doi: 10.1371/journal.pone.0023381 (PMC3154943; doi:10.1371/journal.pone.0023381)

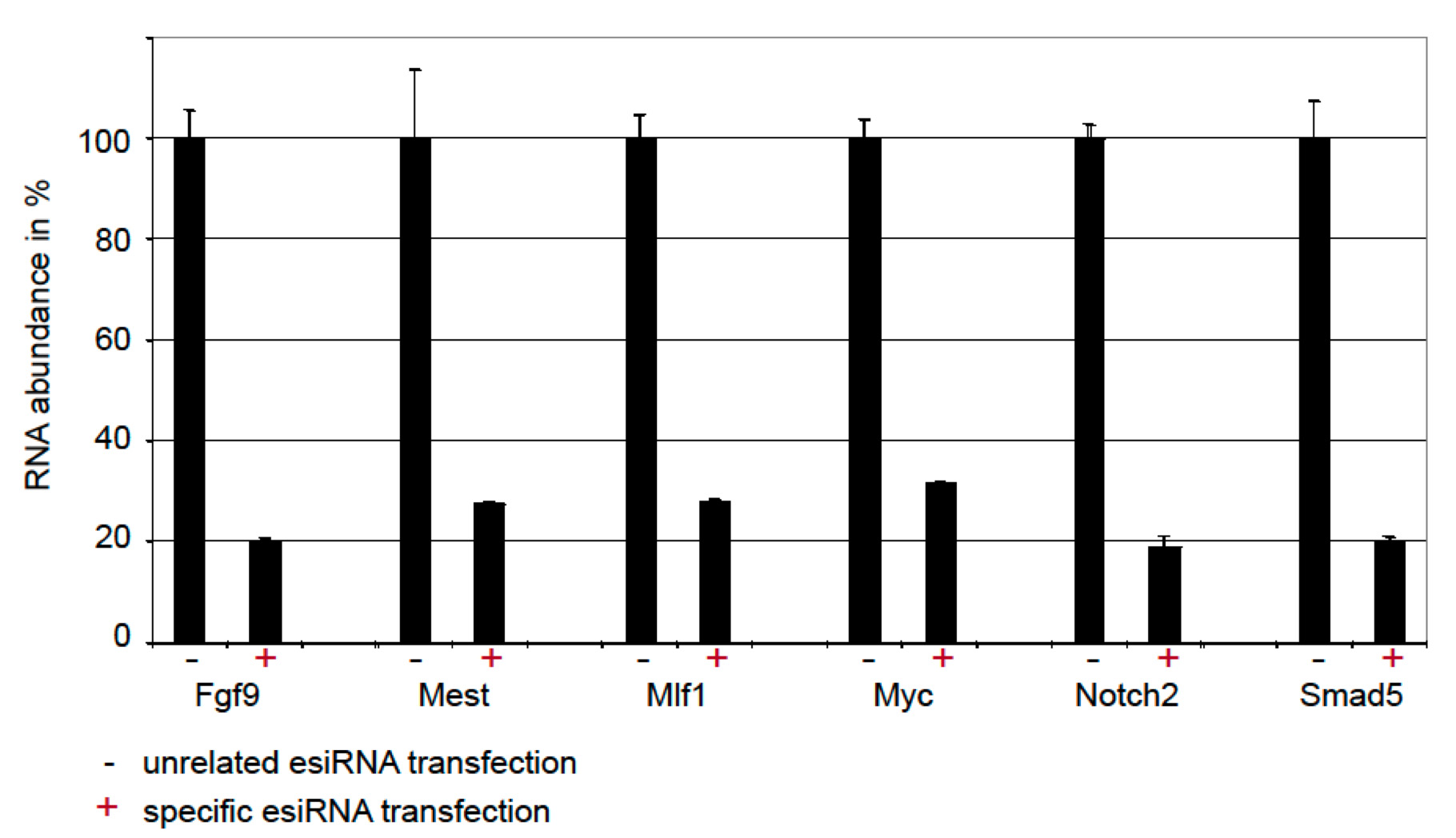

Supplement: Figure S1 — esiRNA treatment induces effective mRNA interference in SW480 cells. SW480 cells were transfected with esiRNAs directed against selected genes or with control esiRNA, and mRNA levels were assessed 48 h after transfection, using qRT-PCR. Expression of experimental genes was normalized versus GAPDH. Residual gene expression after specific esiRNA treatment is given relative to control transfections. Bars indicate mean of three technical replicates and standard error. (TIF) [file pone.0023381.s001.tif]

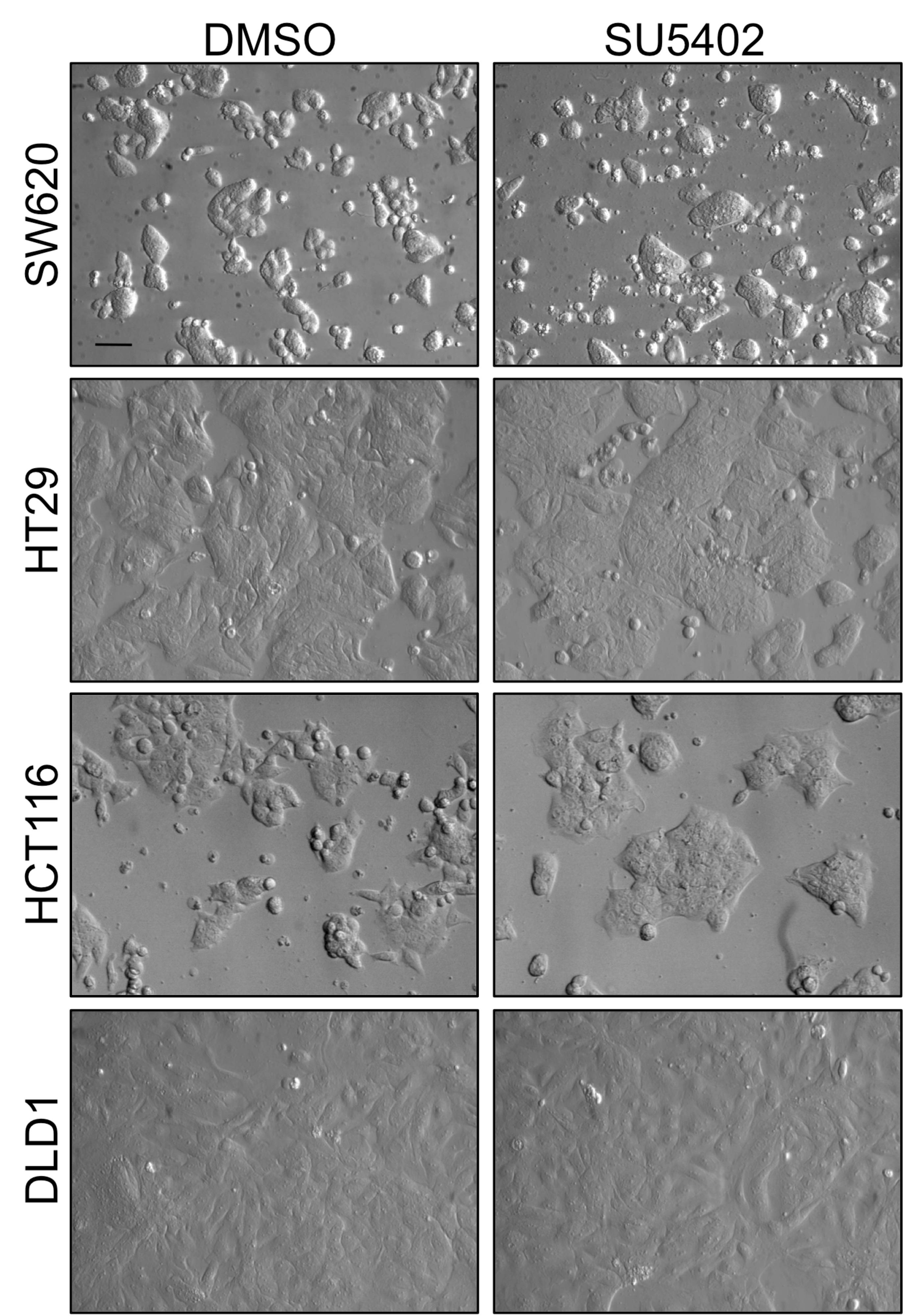

Supplement: Figure S2 — Phenotypic analysis of SW480 and HCT116 colon cancer cells after FGF9 or FGFR3 mRNA interference. A RNA interference with FGF9 cells leads to loss of spindle form and increased membraneous E-cadherin staining in SW480, confirming the screening result (see Fig. 1B). Silencing of FGFR3 does not induce a visible phenotype in SW480. B Interference with FGFR3, but not with FGF9, results in loss of spindle form in HCT116 (see Bright Field), however not to an increase in membraneous E-cadherin. SW480 and HCT116 were transfected with Dharmacon smart pool siRNAs, according to manufacturer's instructions, using DharmaFECT1 and a final concentration of 25 nM siRNA. Scale bars 50 µm. (TIF) [file pone.0023381.s002.tif]

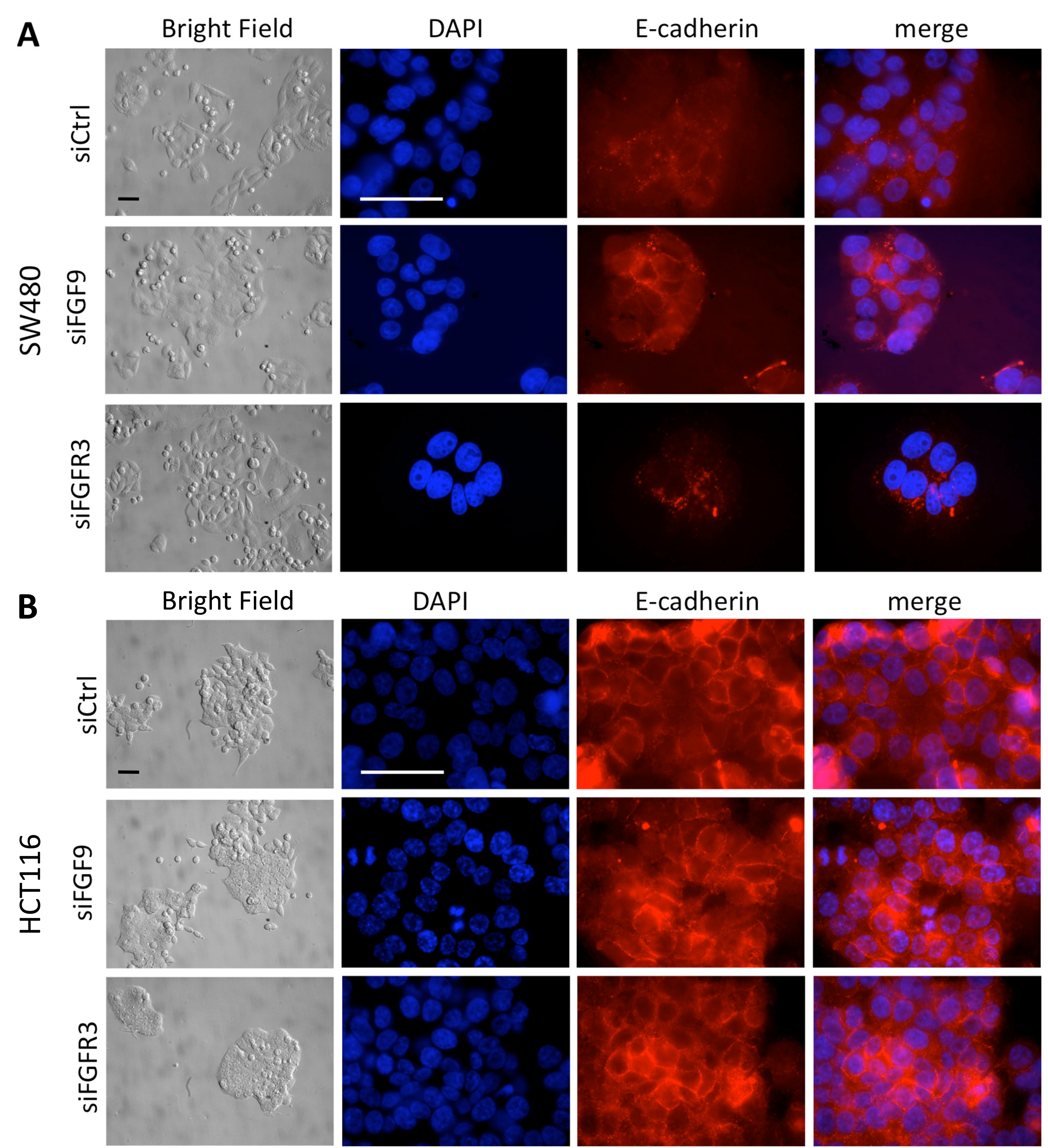

Supplement: Figure S3 — Multiple colon cancer cell lines re-epithelialize upon FGF receptor inhibition. Phenotypes of a panel of colon cancer cell lines after FGF receptor inhibition, using SU5402. SW620, HT29 and HCT116 cells show aspects of re-epithelialization, such as loss of spindle form or disappearance of visible cell-cell contacts. In contrast, DLD1 (lower panels), WiDr, Caco2 and Cx1 cells (not shown) do not show visible phenotypic alterations after inhibitor treatment. Images were taken 72 h after start of the treatment, as in Material and Methods. Scale bar represents 50 µm. (TIF) [file pone.0023381.s003.tif]

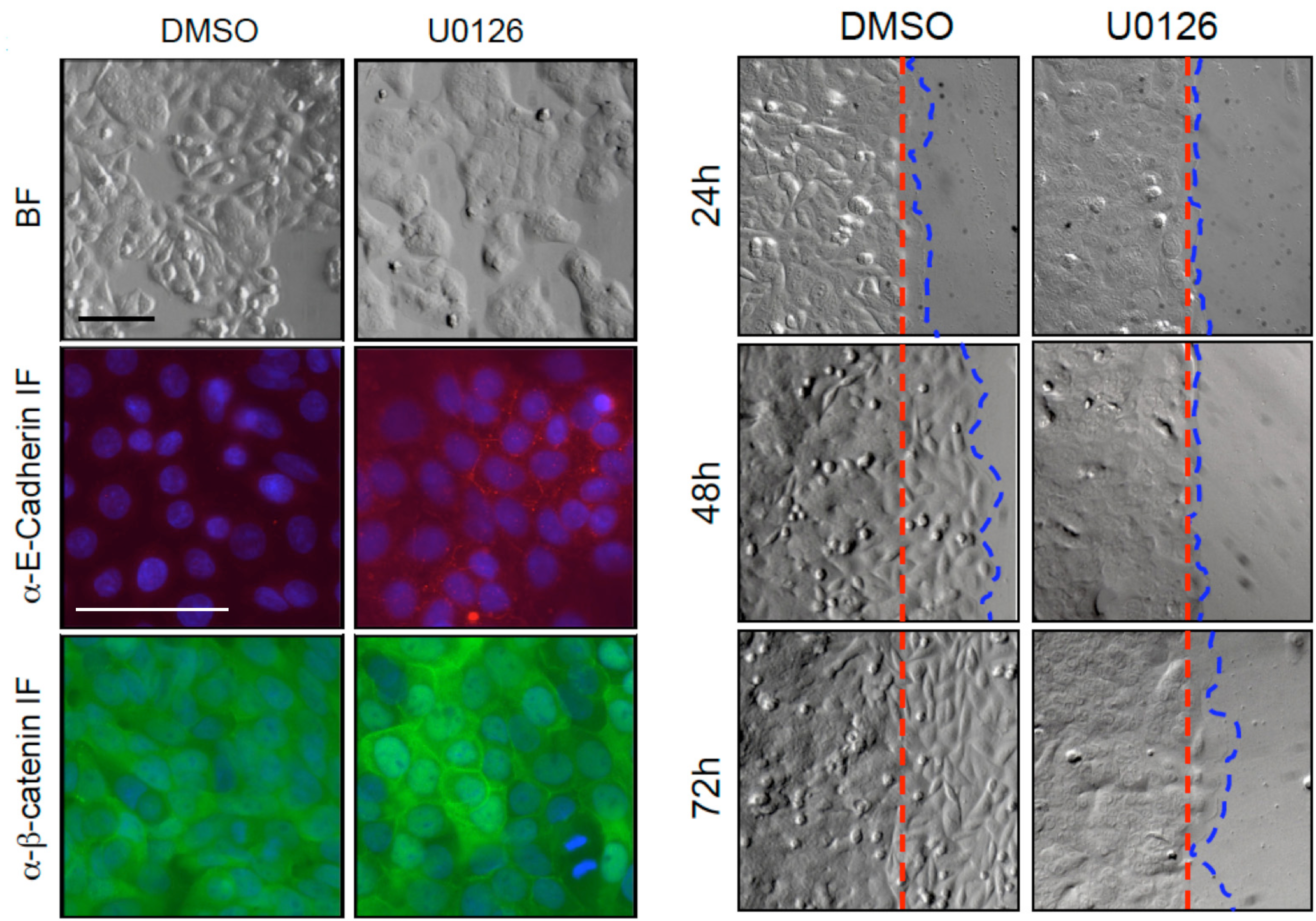

Supplement: Figure S4 — Inhibition of MEK1/2 by U0126 induces re-epithelialization and blocks cell motility in SW480 cells. Left: Phenotypic switch of SW480 cells upon MEK1/2 inhibition. Top to bottom: Cell morphology in phase contrast, immunofluorescence of E-cadherin and b-Catenin, which are components of adherens junctions. Photos were taken 72 h after start of the treatment. Right: Scratch-wound motility assay upon MEK1/2 inhibition. SW480 cells were grown to confluence, and scratches were induced 24 h after start of inhibitor treatment. Photos show phase-contrast images of the scratch edge and were taken 24, 48 and 72 hour after scratching. Red line indicates scratch edge, blue line indicates cell migration front. Scale bars represent 50 µm. (TIF) [file pone.0023381.s004.tif]

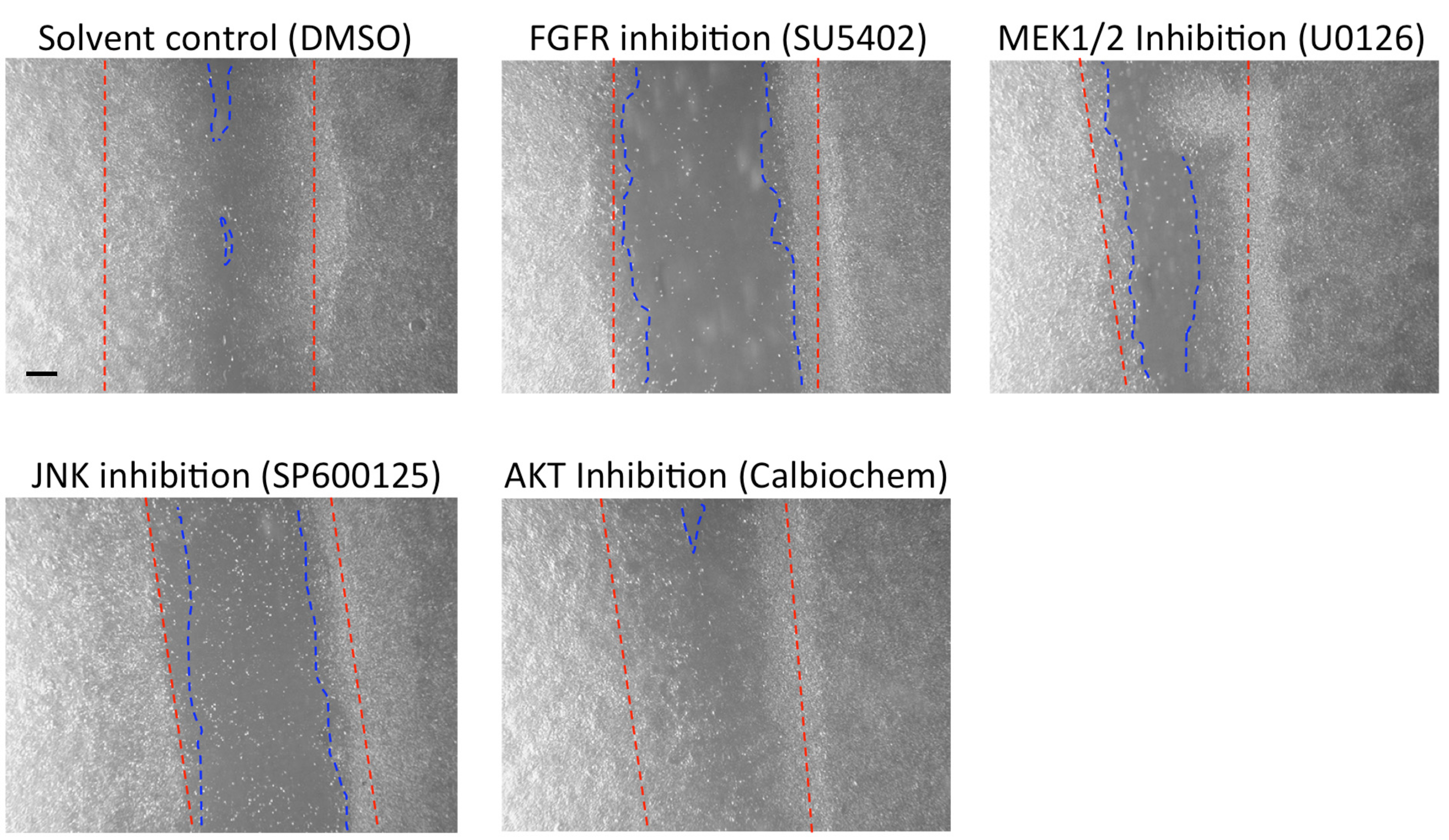

Supplement: Figure S5 — Phenotypic effects of inhibitor treatment in HCT116 colon cancer cells. Scratch-wound assays were performed upon inhibition of FGF receptors, MEK1/2, JNK or AKT, as indicated. HCT116 cells were grown to confluence, and scratches were induced 24 h after start of treatment. Solvent control-treated cells, as well as AKT- inhibitor-treated cells close the scratch wound, whereas FGFR, MEK1/2 or JNK inhibitor treatments lead to a delay in scratch wound closure. Inhibition of PI3K leads to marked loss of cells at the scratch edge (not shown). Bright field images of the scratch were taken 72 hour after scratching. Red line indicates scratch edge, blue line indicates cell migration front. Scale bar represents 100 µm. (TIF) [file pone.0023381.s005.tif]

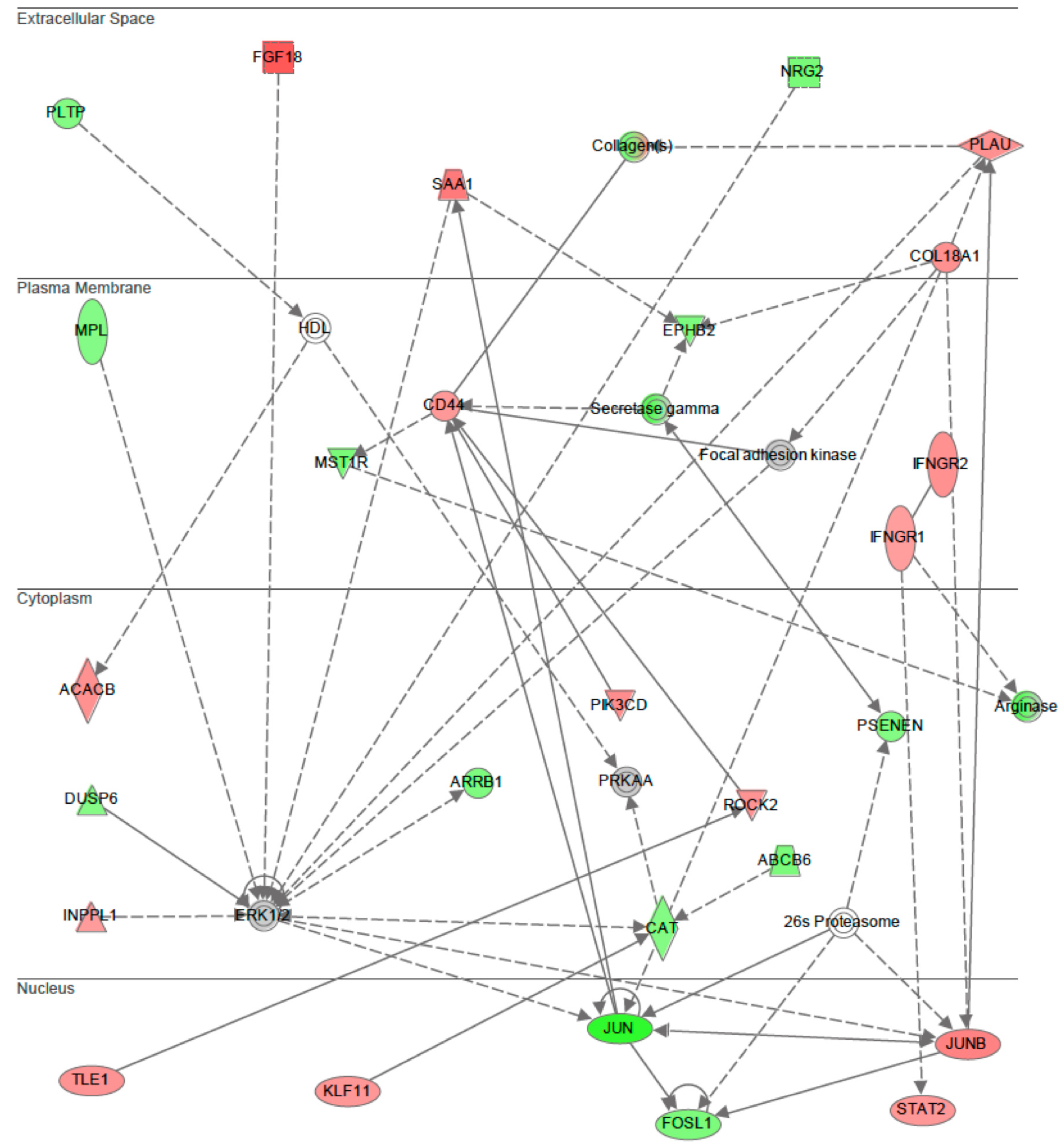

Supplement: Figure S6 — Ingenuity pathway analysis identifies a network implicated in cell motility, which is enriched in FGF-regulated genes. Green: downregulated genes after SU5402 treatment, red: upregulated genes after SU5402 treatment. Grey: non-regulated genes within the network. Network was derived from genes deregulated (>1.3-fold, p<0.05) in expression profiles from biological triplicates of SU5402-treated SW480 cells and solvent controls. Profiles were normalized in Illumina Genome Studio using cubic spline normalization and background subtraction. Absent and marginally expressed genes were removed before analysis. (TIF) [file pone.0023381.s006.tif]

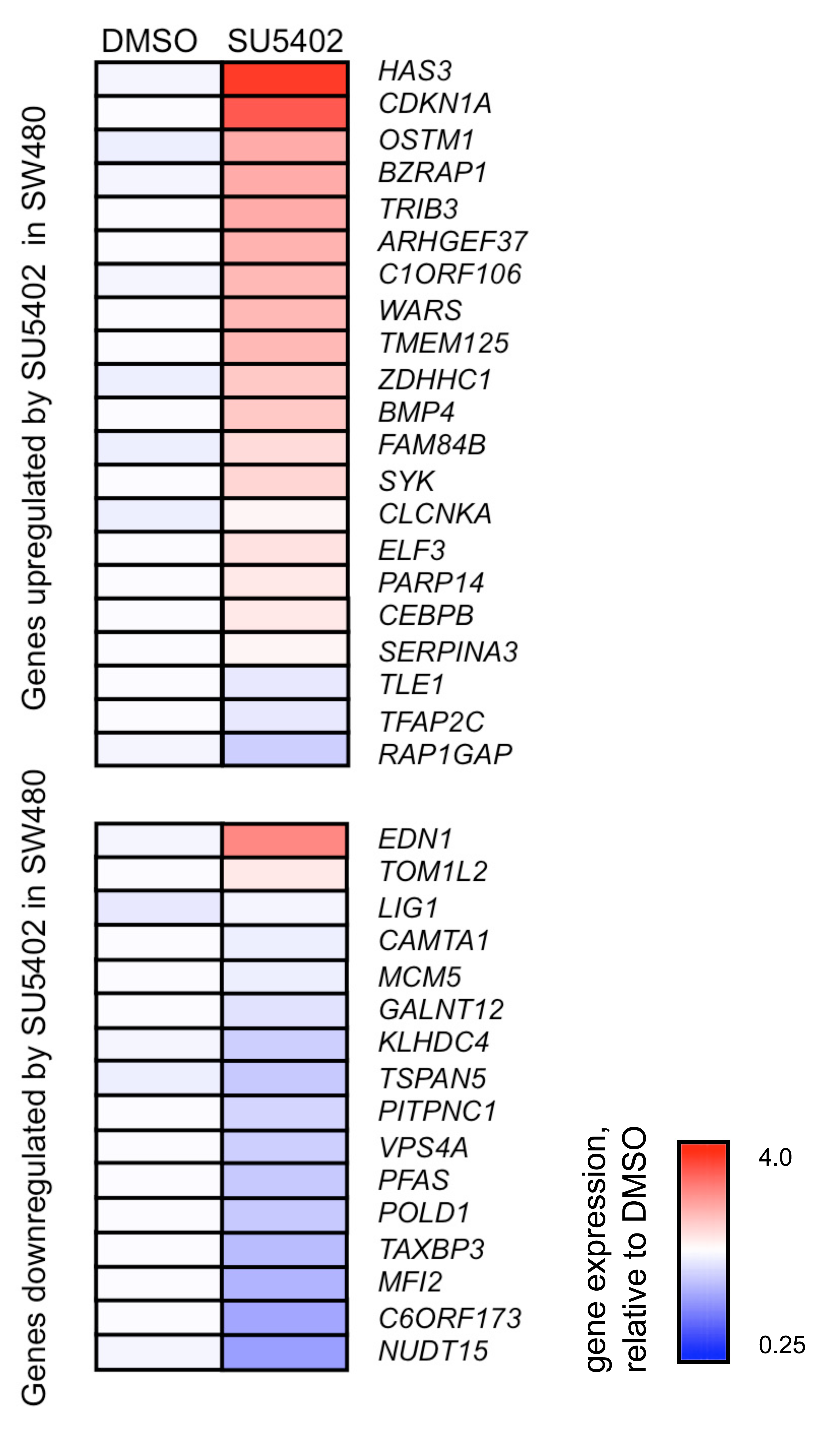

Supplement: Figure S7 — An SW480-derived gene expression program associated with epithelial and mesenchymal morphology is also modulated in HCT116 cells. Analysis of gene expression in HCT116, 24 h after treatment with SU5402 or in solvent control (DMSO). Figure shows genes whose expression has previously been identified to correlate with mesenchymal or epithelial phenotypes in SW480 cells (see Fig. 5A). Genes that are not expressed in HCT116 were removed. Gene expression profiles were assessed using Illumina Sentrix Hu8-v2 bead chips according to manufacturer's instructions. Data was processed from biological duplicates using Illumina Genome Studio, as detailed in the Material and Methods section. Red indicates high expression; blue low expression and white average expression. (TIF) [file pone.0023381.s007.tif]

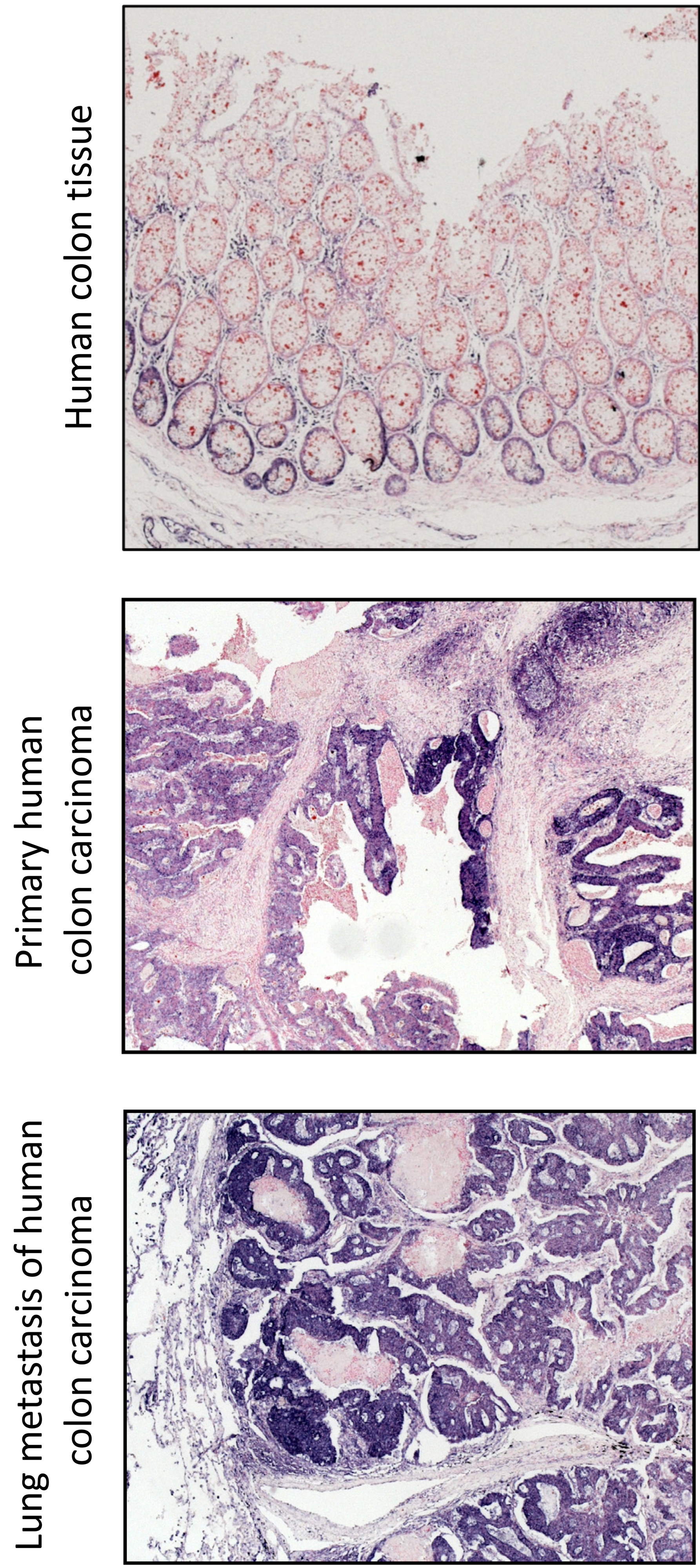

Supplement: Figure S8 — TCF7L1 expression is delocalized and upregulated in human colon carcinoma. Expression of TCF7L1 in normal human colon (upper panel), primary human colon carcinoma (middle panel), and lung metastasis of human colon carcinoma (lower panel), as assessed by RNA in-situ hybridization. Negative control hybridization (using an unrelated asRNA probe of similar size and CG content) did not produce a signal (not shown). (TIF) [file pone.0023381.s008.tif]
